# Cluster analysis of social determinants of health and HIV/AIDS knowledge among Peruvian youths using Kohonen’s self-organized maps: a data-exploration study based on a Demographic and health survey

**DOI:** 10.1080/16549716.2024.2438070

**Published:** 2025-01-17

**Authors:** Alejandro Aybar-Flores, Alvaro Talavera, Elizabeth Espinoza-Portilla

**Affiliations:** a Department of Engineering, Universidad del Pacifico, Lima, Peru; b Faculty of Health Sciences, School of Medicine, Universidad Continental, Lima, Peru

**Keywords:** HIV/AIDS, social determinants of health, unsupervised learning, Kohonen, self-organizing maps, agglomerative hierarchical clustering, youth, Peru

## Abstract

**Background:**

Human immunodeficiency virus (HIV) and acquired immunodeficiency syndrome (AIDS) have evolved into a global development burden, with nearly 40 million infections and 25 million deaths. Compared to other age groups, youth have increased risks of contracting the disease due to social and health structural factors; thus, additional efforts are needed to effectively tackle the challenges associated with this age group. Epidemiological studies employing unsupervised learning techniques are essential for shaping public health policies.

**Objective:**

This study aimed to describe the Peruvian youth population based on their sociodemographic, health, and economic characteristics using an unsupervised learning approach through the development of a neural network model based on Kohonen’s self-organizing maps (SOMs), allowing the identification of social profiles in the study population.

**Methods:**

This quantitative study used data from the 2019 Peruvian Demographic and Family Health Survey. An SOM network model for clustering individuals with similar attributes and clustering prototype vectors based on the agglomerative hierarchical clustering (AHC) method and their visualization on an SOM was applied to the study sample.

**Results:**

Clustering of prototype vectors yielded four clusters, each of which represented a profile of Peruvian youths based on their knowledge of HIV/AIDS and structural health determinants.

**Conclusions:**

Kohonen’s neural networks allowed the identification of patterns and behaviors among youths in Peru, quantifying and characterizing the four social clusters regarding HIV/AIDS and their social determinants. Kohonen’s maps may benefit healthcare professionals and policymakers by offering a useful method for tailoring interventions and policies based on the detected profiles, thereby enhancing the visibility of these focal points at the national level.

## Paper context

- **Main findings:** This study identified four social clusters among Peruvian youths using Kohonen’s self-organizing maps, revealing disparities in HIV/AIDS knowledge linked to socioeconomic and demographic factors, with greater awareness in urban areas and significant gaps in rural and disadvantaged regions.
- **Added knowledge:** This research pioneers the use of Kohonen’s SOM in Peru to cluster populations by socioeconomic and health factors, uncovering subgroups, and demonstrating the effectiveness of the method in capturing complex patterns of health knowledge distribution often overlooked by traditional statistical techniques.
- **Global health impact for policy and action:** The findings inform targeted public health policies by characterizing social clusters, thereby supporting tailored HIV/AIDS education, improving resource allocation, and enhancing the effectiveness of national health initiatives for specific population segments.

## Background

Since their emergence 25 years ago, human immunodeficiency virus (HIV) and acquired immunodeficiency syndrome (AIDS) have evolved from a public health concern to a global development burden, with a daily incidence of approximately 12,000 new cases and 500 transmissions occurring per hour worldwide [1,2].

Given these circumstances, youth is considered a demographic group at risk in global HIV prevention programs [3]; even with a 46% reduction in the rate of new infections in 2019 compared to previous years, 5.1 million individuals were living with HIV in 2019 [4]. In that sense, reduction in the rate of new infections varies across countries, with some countries showing notable decreases in infections among the youth many other low- and middle-income countries showing only modest reductions [5]. Additional efforts are required to address the structural factors that increase youth vulnerability, such as sex-based violence and inequalities, poverty, discrimination, and inadequate sexual education programs [5].

In this regard, Peru recognized the need for strategies to halt HIV transmission as the first cases emerged, leading to the implementation of prevention and control measures. Intervention strategies began in 1988 with the Special AIDS Control Program (PECOS), which focused on prevention, until it was replaced by the Program for the Control of Sexually Transmitted Diseases and AIDS (PROCETS) in 1996, which prioritized patient care and introduced the CONTRASIDA Law to address HIV/AIDS and STIs. Despite these efforts, challenges in reporting and coordination persisted [6]. In 2004, the United Nations identified factors such as lack of contraception and unsafe sex as drivers of HIV, prompting Peru to strengthen sexual health education and launch Highly Active Antiretroviral Therapy” (TARGA), which benefited over 9,000 people by 2006 [7]. The 2007–2011 multisectoral plan bolstered prevention efforts, and in 2013, Peru adopted international guidelines to improve HIV care based on the Cascade of HIV Care and the 90-90-90 objectives from UNAIDS. By 2018, pre-exposure prophylaxis (PrEP) had become a part of the national strategy to protect high-risk populations [7]. In 2023, the Technical Health Standard for Combined HIV Prevention was implemented, integrating biomedical, behavioral, and sociocultural measures to reduce transmission among high-risk groups [8].

Despite these efforts, public interventions have not sufficiently reduced new infections, partly because of ongoing issues such as limited community participation, inadequate management of communication strategies for youths and key populations, low political prioritization of prevention, health services not adapted to specific cohorts, treatment distribution barriers, and stigma toward key populations [9]. Additionally, recent analyses have shown that trends in Peru follow global patterns, highlighting the significant risks and ongoing challenges among youths [10].

The youth in Peru (defined as those aged 15–29 years) represent 24.7% of the total population, which is slightly less than the 0–14 years age group, which comprises 25.8% of the total population [11]. Between 2000 and 2020, Peru recorded 113,807 new HIV cases and 32,128 AIDS cases, with over half of them involving youths older than 20 years. This group accounted for 67.8% of HIV cases and 64.8% of AIDS cases, with a marked sex disparity: four men for every woman with HIV and five men for every woman with AIDS [6]. The highest transmission rates occurred among youths aged 15–24 years, and primary mode of transmission was sexual contact. Although the most affected age group five years ago was 25–29 years, there has been a recent rise in cases among individuals aged 15–21 years [6]. Mortality rates due to HIV/AIDS are 4% per 100,000 among youths, 10% per 100,000 among adults, and <2% per 100,000 among children and adolescents. Risky behaviors, such as early unprotected sex, multiple partners, and substance use, continue to drive HIV spread among youths [6]. Additional factors, including migration, wealth group, limited access to sexual education, media influence, and reluctance to promote healthy practices, also affect sexual behavior and increase risk [6]. Addressing these challenges requires tailored strategies to meet the specific needs of Peruvian youths and risk factors for HIV spread.

Currently, traditional statistical approaches for studying the HIV/AIDS epidemic lack insight into the variation in HIV knowledge and sociobehavioral characteristics within populations [12], often offering only generic observations of the country’s perspective based on variables that were usually analyzed broadly. Therefore, for a more comprehensive understanding of the HIV/AIDS situation, emphasis should be placed on examining the characteristics of the youth population, considering health and socioeconomic factors [12]. Using unsupervised learning, an approach in which algorithms identify patterns in data without labelled inputs, epidemiological studies have gained prominence in designing effective public health policies by monitoring infection trends and identifying influential factors for disease control and long-term reduction [13]. Thus, unsupervised techniques such as cluster analysis offer a means of categorizing individuals into homogeneous groups, leveraging their health and sociodemographic features, among other factors, and potentially explaining disparities within a country’s population [14]. Recent studies have suggested that cluster analysis reveals hidden subgroups with distinct factors and HIV risk levels, aiding targeted interventions [12,15]. Identifying risk profiles through cluster analysis can inform tailored interventions for HIV prevention [16].

In that sense, neural networks, particularly self-organizing maps (SOMs) by Kohonen, have facilitated the resolution of intricate social problems, serving as a method for effective data analysis, as they can group individuals exhibiting similar characteristics and provide an easy way to visualize these characteristics within each cluster [17,18]. The superiority of SOM over other methods lies in its capacity to project and visualize multidimensional data, making it a powerful tool for identifying clusters and understanding the impact of independent factors [19,20]. In particular, SOM allows public health managers to determine whether interventions from strategic sectors are necessary and guide public health endeavors to optimize HIV prevention and testing in low- and middle-income countries [21,22]. In Peru, the application of SOM is particularly relevant, as it can reveal underlying risk profiles within the youth population that traditional analyses might overlook [12]. By employing SOM, we can uncover specific target areas for intervention and provide a more focused approach to public health initiatives in the country.

Nonetheless, to the best of our knowledge, no study has been conducted in Peru to explore the applicability of Kohonen’s neural networks for clustering key populations. Robust research aimed at developing unsupervised learning methods for categorizing individuals according to their socioeconomic and health characteristics is lacking. Our study aimed to address this issue by utilizing Kohonen’s Self-Organizing Maps. This approach uses representative data to evaluate variable importance and cluster heterogeneity. Hence, this study explored the potential of SOM as an effective approach for defining social clusters, enhancing public interventions, and quantifying and characterizing agglomerations within Peru. This approach would allow the Peruvian government to identify challenges within the target population to effectively develop targeted interventions for youth, a demographic currently at a higher risk than others, and improve the country’s public health situation.

## Methods

### Study sample

Data used for the analysis presented in this paper were drawn from the Peruvian Demographic and Family Health Survey (DHS) [23]. The DHS is a research initiative managed by Peru’s National Institute of Statistics and Informatics that provides updated information on demographic dynamics, as well as data on the status and factors associated with non-communicable and communicable diseases [23].

The sampling frame for selection is based on statistical and cartographic information from the 2007 National Population and Housing Census and the Household Targeting System (SISFOH) Update of 2012–2013 [23]. The sample has a two-stage, balanced, stratified, and independent probabilistic design at the departmental level, covering both urban and rural areas to provide nationally representative estimates [23]. The data analyzed included 36,760 households: 14780 from department capitals and Lima districts, 9,320 from other urban areas, and 12,660 from rural areas. The study targeted the usual residents of private households aged ≥15 years who stayed in the household the night before the survey. Data were collected through direct interviews conducted by trained personnel using mobile devices [23].

### Study design and procedures

**Figure 1.**
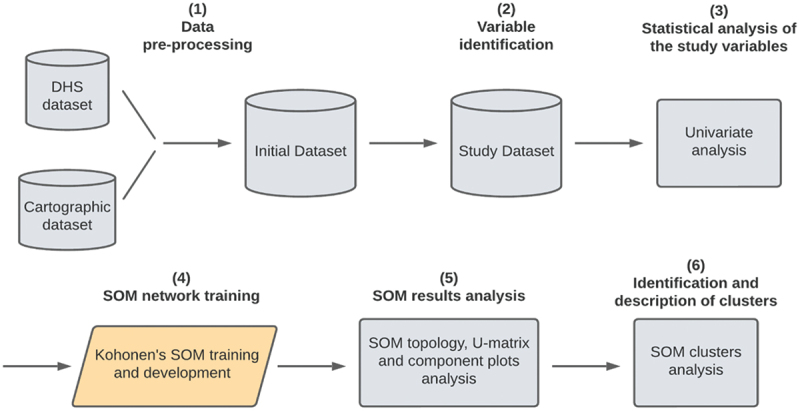
SOM clustering methodology.

The proposed methodology is depicted in Figure 1, as follows:

#### Database pre-processing

In the unification of the study’s database, crucial study indicators were present in the CSALUD01 module, which encompasses the health factors and conditions of individuals aged 15 years and older in the DHS. The data include various conditions, risk factors, perception, and knowledge of communicable and non-communicable diseases, mental health, and so on. Additional modules such as Household, Individuals, and Women were necessary to complete and filter the database for proper survey analysis [24].

**Figure 2.**
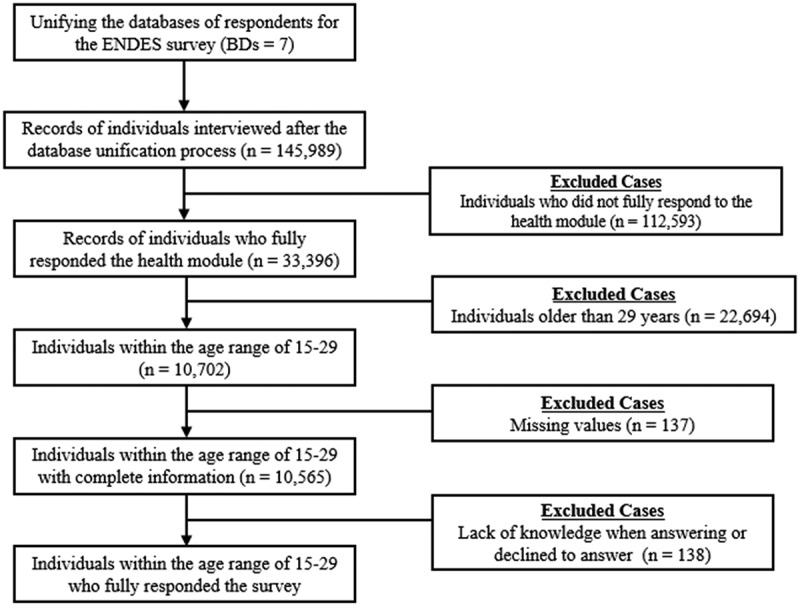
Flowchart of the study selection procedure.

Following the sample preparation procedure defined in Figure 2, after unifying the independent databases into a consolidated DHS dataset, a filtering stage ensues, involving four exclusion criteria.

**Figure 3.**
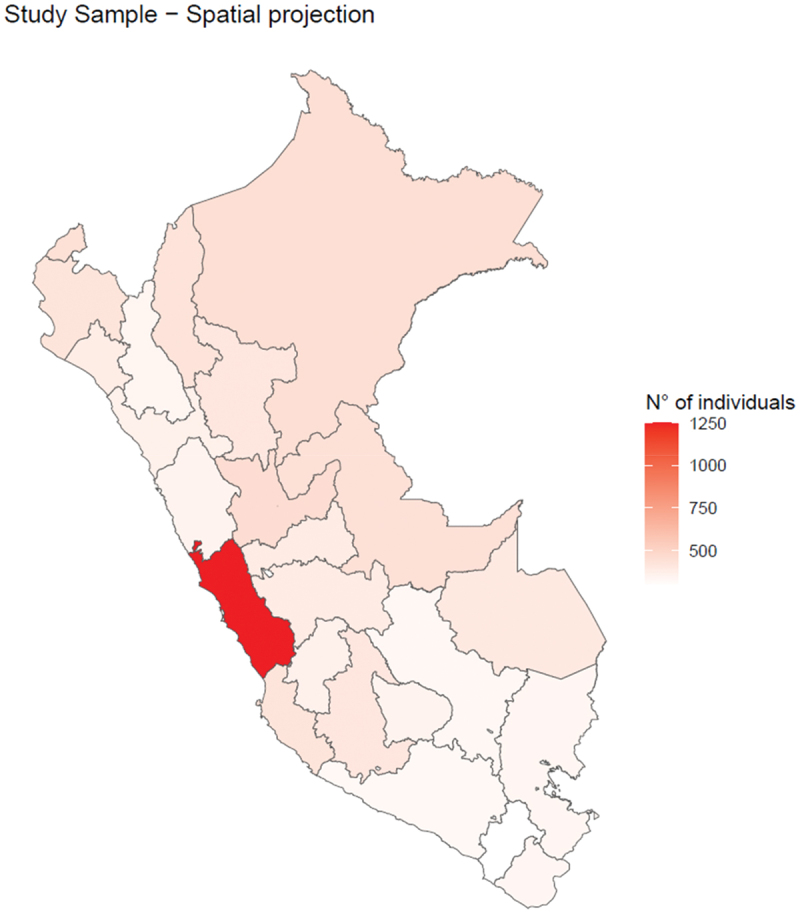
Distribution of the youth population interviewed in Peru.

First, individuals who did not fully respond to the health survey were excluded, reducing the sample from 145,989 to 33,396 respondents aged ≥15 years, as the analysis focused on the health data of individuals in this age group who had properly documented information. Second, individuals >29 years were removed to include only the youth population (ages 15–29 years) in Peru, narrowing the sample to 10,702. After excluding 137 patients with missing data, the dataset was reduced to 10,565. Finally, respondents who did not answer or declined to respond to specific survey questions were excluded, resulting in 138 additional exclusions and a final sample size of 10,427 individuals. Figure 3 presents the distribution of the youth sample.

The dataset had redundant features, such as identifiers created by merging previous modules and characteristics that were not part of the study focus. These features were excluded, leaving 15 variables for the analysis.

#### Variable identification

After the sample preparation and feature selection procedures, the variables used in the study and their descriptions and values are summarized in Table 1.

**Table 1.** Definition and description of the variables involved in the self-organizing map.

Variable	Definition
HIV/AIDS knowledge	Knowledge of ways to avoid HIV/AIDS, perception of infection risk, and the social aspects of prevention and mitigation
Sex	Biological sex of the interviewee
Residence area	Zone boundaries of the respondent’s household
Educational level	Highest level of education attained by the respondent
Ethnicity	Ethnicity that the respondent identifies with based on culture, customs, and ancestry
Primary language	Language the respondent learned in their early years
Heard of HIV/AIDS	Exposure to information related to HIV/AIDS
Insurance status	Whether the respondent has health insurance coverage
Engagement in care	Whether the respondent received treatment from a healthcare professional for health issues in the last 12 months
HIV/AIDS testing	Whether the respondent underwent HIV/AIDS testing in the last 12 months
Age	Age group of the respondent
Region of residence	Region of the respondent’s residence
Mass media access	Access to mass media (includes radio, TV, and internet)
Sex of household head	Biological sex of the head of the respondent’s household
Wealth Index	Wealth group to which the respondent belongs

Based on Table 1, the sociodemographic characteristics of the respondents included individual-level factors such as sex, age (segmented into three age ranges: 15–19, 20–24, and 25–29, reflecting the age groups used for studying the youth population in the country [11]), region of residence, highest level of education, and the sex of the household head, which provide an important context for understanding individual circumstances.

Individual cultural factors, that may shape interactions with health information and influence overall health outcomes, include respondents’ self-perceived ethnicity and primary language.

Social context factors include the wealth index, area of residence and access to mass media. The wealth index was developed by researchers from Macro International Inc. and the World Bank and applied in Peru to assess the wealth group to which identified households belong [23]. This index assigns wealth levels to households based on characteristics such as housing, access to services, ownership of durable goods, cooking fuel type, and household density. Each household receives a score using the principal components method, which evaluates the various characteristics considered, allowing for the combination of these scores into a comprehensive index that summarizes the wealth level of the households. This index provides a thorough assessment of an individual’s socio-economic status without relying on income or expenditure data, which may not accurately capture the complexities of household wealth and are not collected by the DHS [23]. According to Peruvian policies [23], this calculated wealth index is used to identify patterns in health and service use, as it reflects the gradient of health outcomes across socio-economic levels. By classifying households into five wealth groups, this index can reveal correlations between socio-economic status and health factors (such as HIV/AIDS knowledge), as disparities in wealth may influence access to information and health resources. This connection is significant in recognizing common or distinct patterns among populations of varying socio-economic status. Moreover, the index serves as a valuable tool for social policy and resource allocation, guiding interventions toward those in dire need while facilitating the identification of at-risk populations [23].

Finally, health and healthcare engagement determinants encompass whether the interviewee possesses knowledge about HIV/AIDS, has health insurance coverage, has received treatment from a healthcare professional for health issues in the last 12 months, has received information about HIV/AIDS, and has recently undergone an HIV screening test within the last 12 months.

HIV/AIDS knowledge is not directly listed in the database but is derived from evaluating responses to related health questions. This knowledge can be defined as a set of information, skills, concepts, and understandings that individuals possess about HIV/AIDS, including its transmission, risk, prevention, and management. It also includes knowledge of specific and programmatic ways to avoid HIV/AIDS, the respondent’s perception of infection risk, and the social aspects of prevention and mitigation. The survey measured this knowledge and classified it as adequate or insufficient through responses to five questions on prevention and misconceptions [23]. We relied on features included in the DHS and validated them for HIV/AIDS knowledge assessment using the guidelines for developing basic indicators for monitoring the political declaration on HIV/AIDS formulated by the Ministry of Health (MINSA) in Peru [23]: 1) QS606 – Do you think people have a lower risk of acquiring the virus that causes HIV/AIDS if they have only one sexual partner who is not infected and who has no other partners? (Yes/No); 2) QS607 – Do you think people who use a condom every time they have sex have a lower risk of acquiring the virus that causes HIV/AIDS? (Yes/No); 3) QS608 – Do you think people who are HIV-positive can appear healthy? (Yes/No); 4) QS609 – Do you think people can acquire HIV/AIDS by hugging, kissing, or touching an infected person? (Yes/No); and 5) QS611 – Do you think people can acquire HIV/AIDS from a mosquito bite or by sharing food or utensils with an infected person? (Yes/No). Knowledge was considered adequate when all five questions were answered correctly; otherwise, it was classified as inadequate [23]. Respondents had to correctly recognize prevention methods such as having one faithful and uninfected partner (Yes to QS606) and condom use (Yes to QS607), and note that infected individuals have a healthy appearance (Yes to QS608), while rejecting false beliefs about transmission through casual contact (No to QS609), insect bites, or sharing food and utensils (No to QS611).

#### Preliminary analysis of study variables

Univariate analysis explores qualitative data, providing descriptions of individual variables that are relevant for inclusion in more advanced tests to facilitate subsequent delineation of the structure and development of further analyses [25].

#### SOM network training

An SOM is a type of neural network designed for clustering and data visualization tasks that employs unsupervised learning to analyze data by recognizing patterns and structures without the need for labelled inputs. This allows the network to effectively group similar data points and provide meaningful visual representations of complex datasets [26]. It also transforms high-dimensional data into a low-dimensional discrete map while preserving the topological relationships among data points [26]. In this study, SOM was used to analyze and cluster individuals based on socioeconomic and health characteristics, identifying patterns across the population.

The SOM training process requires the definition of both the topological structure of the map and the criteria for model initialization and development [27]. For the map structure, we employed a two-dimensional (2D) square lattice of neurons with a hexagonal arrangement for smoother data mapping and equidistant neighboring neurons. Grid sizes of 10 × 10 (100 nodes), 15 × 15 (225 nodes), 20 × 20 (400 nodes), and 25 × 25 (625 nodes) were evaluated to determine the optimal configuration for cluster visualization.

The training quality of the map was assessed using several standard evaluation metrics [28]: quantization error measures the average distance between each data point and its closest map unit, with smaller values indicating better model fit; percentage of explained variance shows how much of data variability is captured by the map, with higher percentages indicating better performance; topographic error evaluates how well the map preserves data structure, reflecting the proportion of cases where the second closest node is not adjacent to the first; and Kaski-Lagus error (K-L) was used to assess the geodesic distance between a data point and the second-best matching unit, providing further insights into map quality. These metrics ensured that the selected SOM configuration accurately reflected the input data [28].

Linear initialization was used to set the initial weight vectors based on the minimum and maximum values of the dataset [29]. This method ensures an efficient learning process. A batch-update algorithm was selected to train the weights, offering superior results by updating the neuron weights based on the entire dataset after each epoch [30]. Training was conducted for 200 epochs [31], which ensured convergence and yielded stable clusters based on neighborhood distance.

#### SOM results analysis

Once the SOM was trained, the best matching units (BMUs) were identified, corresponding to neurons that best represent the data points assigned to them [32]. In the resulting SOM visualization, each hexagonal cell represents a neuron and the number of hits (or BMUs) it receives, offering a clear representation of how individuals are grouped.

Visualization tools such as component planes and U-matrices were used to explore and interpret the structure of the resulting clusters [33]. Component planes provide insights into how individual variables influence the formation of clusters, showing the variation in each feature across the map [32]. The unified distance matrix (U-matrix) displays the distances between adjacent neurons, aiding the identification of well-separated clusters [32]. Darker areas on the U-matrix indicate larger distances between neurons, suggesting that the observations in those areas are more distinct, whereas lighter areas indicate greater similarity between neighboring neurons.

#### Identification and description of clusters

We used the U-matrix to visualize distance relationships and guide the identification of clusters [34]. By examining the matrix, we distinguished areas where neurons were closely related (indicating similar individuals) from those where they were apart (representing different groups) [32,34].

To formalize the identification of clusters, we applied agglomerative hierarchical clustering (AHC). This technique groups SOM neurons based on the similarity of their weight vectors, further refining cluster boundaries. AHC is commonly used with SOMs because it reveals hierarchical relationships among units, which is useful for interpreting the underlying structure of the data.

The optimal number of clusters was determined by testing different values of k (from 2 to 10 clusters), and the limit of 10 clusters was considered a reasonable number in the search for profiles of sociodemographic, health, and economic features because of the large number of vectors in the training dataset [35]. Various clustering validity indices (CVIs) were calculated for each k-value using the NbClust package [36]. These indices evaluate the quality of clusters by measuring their compactness and separation. In addition, a scree plot was employed to visually determine the optimal number of clusters by analyzing the variance explained by each cluster configuration. The inflection point on the curve, where the rate of variance reduction slows, indicates the optimal configuration. By combining the results from the CVIs and the scree plot, we ensured a robust and accurate selection of the final number of clusters.

Finally, to explore the distribution of the identified clusters within the Peruvian territory, a spatial projection of individual distribution will be conducted across Peru’s administrative divisions comprising 25 regions [37]. This data integration with governmental cartographic information will aid in assessing the cluster distribution within Peruvian territory and its implications for public policies.

### Statistical analysis

The SOM network model for clustering individuals with similar attributes in the database, the clustering of prototype vectors based on the AHC method, and their visualization on the SOM were accomplished with Kohonen [38] and NbClust [36] packages using RStudio software. The geographic representation and concentration of clusters in Peru were determined using Python.

## Results

### Descriptive statistics of study features

Considering the sociodemographic, economic, and health factors in the analysis cohort, Table 2 displays the counts and proportions of each category pertaining to each study variable.

**Table 2.** Descriptive statistics of the factors from the 2019 DHS within the study.

Variable	Frequency (n)	Proportion (%)
**HIV/AIDS knowledge**		
No	6887	66
Yes	3540	34
**Sex**		
Female	6399	61
Male	4028	39
**Residence area**		
Rural	3315	32
Urban	7112	68
**Wealth Index**		
Lowest index	3165	30
Second lowest index	3062	29
Middle index	2053	20
Second highest index	1363	13
Highest index	784	8
**Region of residence**		
Metropolitan Lima^c^	1189	11
Coast	3037	29
Sierra	3501	34
Jungle	2700	26
**Age**		
15–19 years	2856	27
20–24 years	3284	31
25–29 years	4287	41
**Education level**		
Illiterate	30	0
Elementary school	991	10
High school	6284	60
Higher education	3122	30
**Ethnicity**		
Native^a^	3576	34
Afro-peruvian	1145	11
Caucasian	592	6
Mixed	4356	42
Other/unaware	758	7
**Heard of HIV/AIDS**		
No	1352	13
Yes	9075	87
**Insurance status**		
No	2690	26
Yes	7737	74
**Engagement in care**		
No	10114	97
Yes	313	3
**HIV/AIDS testing**		
No	7734	74
Yes	2693	26
**Mass media access**		
No	1094	11
Yes	9333	89
**Sex of household head**		
Female	2899	28
Male	7528	72
**Primary language**		
Native^b^	1852	18
Spanish	8565	82
Foreign language	10	0

^a^Quechua, Aymara, native to the Amazon, or other indigenous people. ^b^Quechua, Aymara, or another native language. ^c^Province of Lima and the Constitutional Province of Callao.

The table reveals trends across various demographic and socioeconomic factors. Most participants (66%) lacked HIV/AIDS knowledge, with a higher representation among women (61%). Urban areas are more densely populated, with 68% of individuals residing in cities. Economic disparities were evident, with nearly 60% classified in either the second lowest or the lowest wealth index. The regional distribution shows Sierra as the most populated area (34%), followed by the coast (29%) and jungle (26%). The age range was skewed toward older individuals, particularly those aged 25–29 years (41%). The highest education level varied, with most participants (60%) having completed only high school, whereas a smaller percentage (30%) had pursued higher education. Ethnic diversity was notable, with native groups comprising 34% of the population, followed by mixed ethnicities comprising 43%. Awareness of HIV/AIDS was notable, with 87% having heard of the disease and only 26% having undergone testing. Access to mass media was high (89%), while the sex of the household head showed male dominance (72%). The primary language showed a Spanish majority (82%), although a minority (18%) spoke native languages. Regarding health coverage, 74% of the population had health insurance. Engagement in healthcare services appeared to be low, with only 3% of the sample having received treatment from a healthcare professional in the past 12 months.

### Training of the self-organizing map

The training utilized 10,467 observations along with 15 independent variables in the Kohonen network. Table 3 presents the SOM model map grids assessing the social determinants of health and HIV/AIDS knowledge among the youth in Peru, along with the quality measures for each set.

**Table 3.** Assessment of training and clustering for each model grid in SOM training.

Model grid	Number of units	Quantization error	Explained variance %	Topographic error	Kaski-Lagus error (K-L)	Empty nodes
10 x 10	100	1.80	75.80	0.30	3.60	0
15 x 15	225	1.50	80.10	0.40	4.20	0
20 x 20	400	1.20	83.10	0.50	4.40	1
25 x 25	625	1.10	85.10	0.40	3.90	4

In the first map with a 10 × 10 grid, the quantization error was 1.80, with an explained variance of 75.80%. The topographic error was 0.30, and the K-L was 3.60. Importantly, this model had no empty nodes. The second map, with 225 units, had a quantization error of 1.50, explained variance of 80.10%, topographic error of 0.40, and K-L of 4.20, with no empty nodes. In the third grid of 400 units, the quantization error was 1.20, the explained variance reached 83.10%, the topographic error was 0.50, and K-L was 4.40, with one empty node. Finally, the fourth model, using a 25 × 25 grid, had a quantization error of 1.10, explained variance of 85.10%, topographic error of 0.40, and K-L of 3.90, containing four empty nodes.

Upon evaluating the quality metrics, the 20 × 20 grid emerged as the most suitable for this study, demonstrating superior performance compared to the 10 × 10 and 15 × 15 grids across the assessed criteria. Although the 25 × 25 grid exhibited better results for some metrics, its higher number of empty nodes suggests that it may be excessive for the sample size. The 20 × 20 grid offers a balanced distribution of elements with only one empty unit, indicating a more effective representation of the data.

### Analysis of the results of the Kohonen self-organizing map

**Figure 4.**
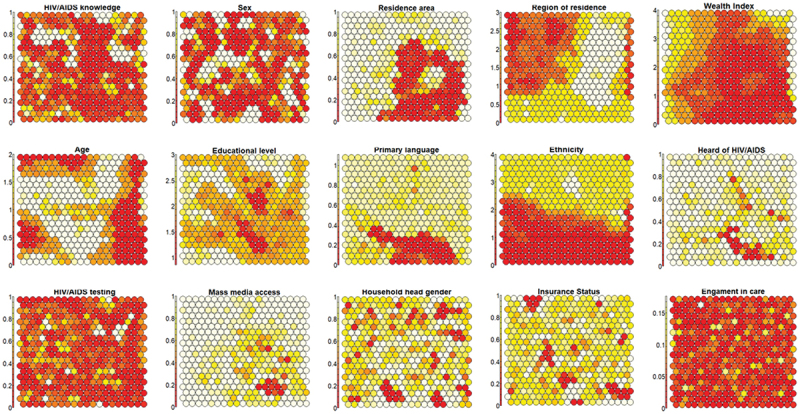
Component planes/plots for the formation of clusters in the SOM network.

The experiments indicated that a 20 × 20 grid was effective in identifying patterns among Peruvians, revealing relationships between features and forming groups based on social characteristics. The component plots shown in Figure 4 separately map each feature of node weights onto the two-dimensional map. These plots represent synthetic versions of the SOM network, revealing the distribution of the vector components of each weight and allowing the detection of associations between variables. Thus, 15 maps arose from demographic and health features, providing an analytical framework for interpreting the relationships among plot properties. The figures are interconnected by position, as a hexagon at a specific spot corresponds to the same map unit.

Through visual analysis of the component plots, the following conclusions can be drawn: (a) ‘Area of residence’ and ‘Wealth index’ are directly related, while ‘Region of residence’ and ‘Wealth index’ are inversely related; (b) ‘Primary tongue’ and ‘Heard about HIV/AIDS’ are directly related, as are ‘Heard about HIV/AIDS’ and ‘Mass media access’; and (c) other features do not exhibit interrelationships.

### Identification and characterization of social clusters of the youth population

**Figure 5.**
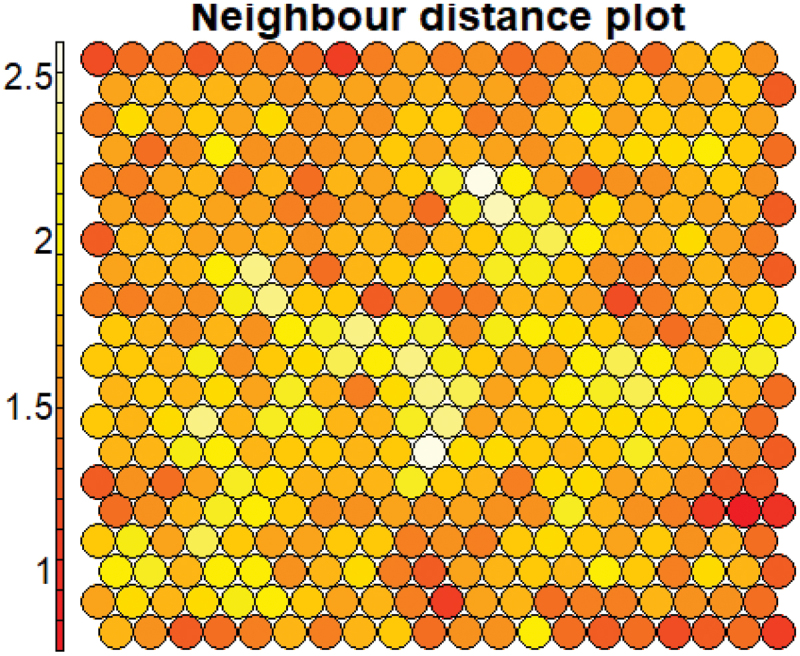
SOM neighbor distribution plot.

Figure 5 presents matrix U, a tool for identifying potential data groupings. The U-matrix map was created by mapping the average distance between each node and its nearest neighbors in a high-dimensional space.

Preliminary manual identification of clusters based on the U-matrix indicated approximately four clusters: a partition at the bottom-right corner, a division in the upper-right corner, a partition at the bottom of the graph, and another extending from the upper-left corner to the center region.

**Figure 6.**
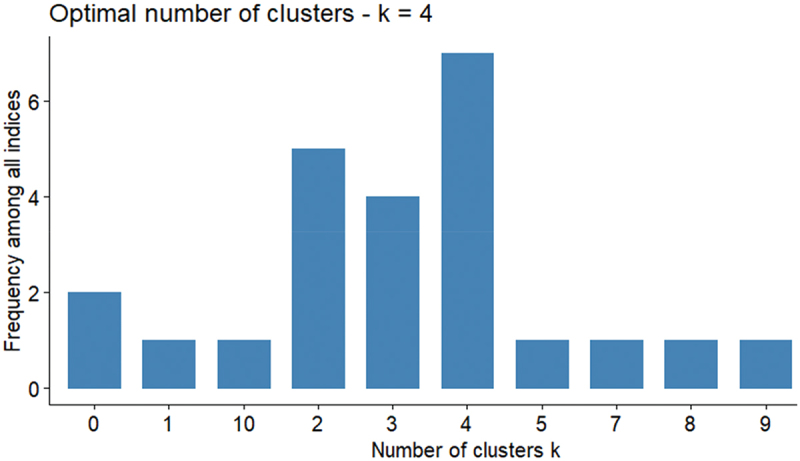
Identification of the optimal number of clusters using CVIs from NbClust.

Although manual identification is helpful, it is insufficient for clearly defining clusters within an SOM. Thus, we applied a clustering method to the prototypes organized in nodes obtained from the SOM model to identify groups with similar sociodemographic, economic, and health characteristics. We employed AHC on the SOM nodes using different CVIs to determine the appropriate number of clusters from k = 2–10. This process, which utilizes a grid of 400 neurons, resulted in nine groupings with various CVIs calculated for each k value tested. The analysis indicated a significant cluster configuration favoring the four clusters, as shown in Figure 6.

*Notes.-* Indices and values supporting k = 4 are as follows: Hartigan (613.60), CCC (108.40), TraceW (2671.70), Rubin (−0.283), Ratwosky (0.15), PtBiserial (0.58), and SDindex (1.30). Higher Hartigan, CCC, and PtBiserial values suggest stronger cluster separation, whereas lower TraceW, Rubin, and SDindex values indicate better compactness and stability.

**Figure 7.**
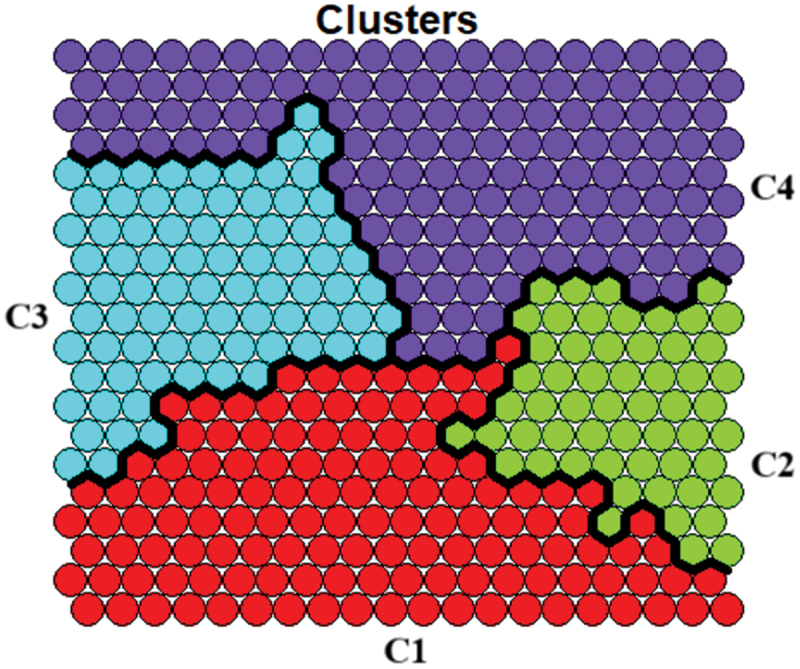
Identification of optimal clusters.

The selection of four clusters allows for a more detailed division, potentially revealing finer patterns and relationships among nodes. This evaluation checks the relatedness of clusters on the map and how interactions manifest within and between clusters. We examined the suggested maps by clustering the SOM nodes using the proposed configuration, as shown in Figure 7.

Analysis of the organized nodes within each cluster reveals contiguous positioning on the map surface, indicating effective data organization. The separation between clusters suggests distinctiveness among identified groups, with minimal variation within each cluster. This high degree of heterogeneity between clusters, coupled with increased consistency within partitions, enhances the distinct profiles within the population. The analysis suggests the benefits of the four-cluster configuration, in which clusters maintain spatial continuity on the map and show greater internal consistency, resulting in a more accurate representation of the data.

**Figure 8.**
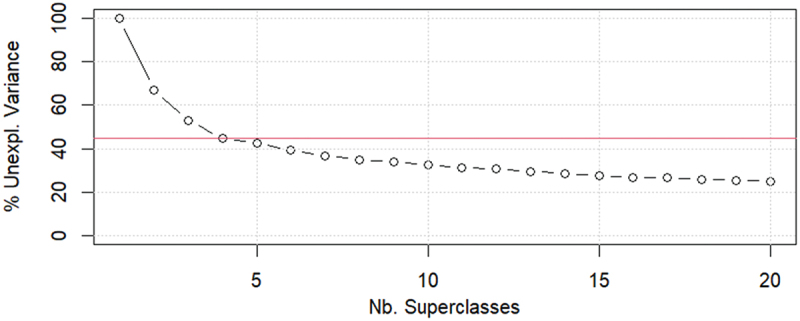
Identification of the optimal number of clusters based on the scree plot analysis.

The optimal number of clusters was further evaluated using the scree plot in Figure 8, which assesses clustering quality by examining the unexplained variance among prototype vectors for different cluster counts. The plot shows the optimal number corresponding to the inflection point on the curve, where the reduction in the unexplained variance diminishes.

According to this analysis, the inflection point occurs at k = 4, indicating that grouping the data into four clusters is the most effective strategy for SOM, balancing distribution, and clustering performance. This choice aligns with CVIs and confirms that the configuration offers a suitable representation of data with manageable variance. Thus, the optimal SOM for this study was a 20 × 20 grid (with 400 units), revealing four distinguishable clusters that align with the AHC results, CVI validation, and scree plot analysis.

Statistical descriptions of the sociodemographic, economic, and health profiles of each cluster are presented in Table 4. Additionally, mean difference tests using the Chi-square or Fisher’s tests were used to assess cluster differentiation across each study feature.

**Table 4.** Socio-demographic, economic, and health characteristics of the social clusters.

	Clusters	C1	C2		C3		C4			
Variable	Categories	n	%	n	%	n	%	n	%	Mean diff. ^b^
HIV/AIDS knowledge	No	2530	75	845	61.00	1481	71.00	2031	57.00	<0.01 ***
	Yes	842	25.00	538	39	615	29	1545	43	
Sex	Female	2210	66	727	53	1279	61	2183	61	<0.01 ***
	Male	1162	34	656	47	817	39	1393	39	
Residence area	Rural	1902	56	56	4	1143	55	214	6	<0.01 ***
	Urban	1470	44	1327	96	953	45	3362	94	
Wealth index	Lowest index	1833	54	23	2	1173	56	136	4	<0.01 ***^a^
	Second lowest index	1212	36	274	20	909	43	667	19	
	Middle index	297	9	524	38	14	1	1218	34	
	Second highest index	30	1	400	29	0	0	933	26	
	Highest index	0	0	162	12	0	0	622	17	
Region of residence	Metropolitan Lima	8	0	334	24	0	0	847	24	<0.01 ***^a^
	Coast	264	8	757	55	75	4	1941	54	
	Sierra	2192	65	234	17	708	34	367	10	
	Jungle	908	27	58	4	1313	63	421	12	
Age, years	15 to 19	894	27	383	28	579	38	1000	28	<0.01 ***
	20 to 24	1068	32	423	31	677	32	1116	31	
	25 to 29	1410	42	577	42	840	40	1460	41	
Education level	Illiterate	15	0	2	0	11	1	2	0	<0.01 ***^a^
	Elementary school	541	16	44	3	333	16	73	2	
	High school	2248	67	718	52	1427	68	1891	53	
	Higher education	568	17	619	45	325	16	1610	45	
Primary language	Native	1493	44	176	13	122	6	61	2	<0.01 ***^a^
	Spanish	1875	56	1207	87	1973	94	3510	98	
	Foreign language	0	0	0	0	1	0	5	0	
Ethnicity	Native	2731	81	845	61	0	0	0	0	<0.01 ***^a^
	Afro-Peruvian	523	16	487	35	134	6	1	0	
	Caucasian	116	3	51	4	170	8	255	7	
	Mixed	2	0	0	0	1458	70	2896	81	
	Other/unaware	0	0	0	0	334	16	424	12	
Insurance status	No	985	29	330	24	530	25	845	24	<0.01 ***
	Yes	2387	71	1053	76	1566	75	2731	76	
Engagement in care	No	3278	97	1336	97	2033	97	3467	97	<0.01 ***
	Yes	94	3	47	3	63	3	109	3	
Heard of HIV/AIDS	No	779	23	85	6	321	15	167	5	<0.01 ***
	Yes	2593	77	1298	94	1775	85	3409	95	
HIV/AIDS testing	No	2564	76	986	71	1587	76	2597	73	<0.01 ***
	Yes	808	24	397	29	509	24	979	27	
Mass media access	No	579	17	47	3	370	18	98	3	<0.01 ***
	Yes	2793	83	1336	97	1726	82	3478	97	
Sex of household head	Female	867	26	430	31	518	25	1084	30	<0.01 ***
	Male	2505	74	953	69	1578	75	2492	70	
	Subtotal ^c^	3372	32	1383	13	2096	20	3576	34	
	Total^d^	10427	100							

***highly significant values *p* < 0.01; ^a^Indicates that Fisher’s mean difference test was performed; ^b^test of mean difference between clusters; ^c^total number of individuals forming part of each cluster and its percentage value; ^d^total sample.

All four clusters exhibit varying degrees of HIV/AIDS knowledge and distinct socioeconomic and health features on a particular level. Additionally, mean difference tests were conducted for each study factor, and each significance value indicates a significant difference between the clusters for each corresponding variable used in the study, suggesting the distinctiveness of the clusters in terms of specific characteristics.

Cluster 1 comprises individuals with relevant knowledge about HIV/AIDS, primarily women residing in urban and rural areas. This group largely belongs to the lowest wealth index group, predominantly in Sierra. The age distribution skews slightly toward older women, with many aged between 25 and 29 years. The educational attainment of this population is generally at the high school level, and a significant portion speaks native languages alongside Spanish. Most women identify as native ethnically. While many have heard of HIV/AIDS (although it is the lowest among all clusters), the testing rates remain low. Media access is widespread and most households are male-headed. Most members have health insurance, but their engagement in care is minimal.

Cluster 2 exhibits a lower level of HIV/AIDS awareness than Cluster 1, with a relatively balanced sex distribution. Almost all members reside in urban areas and typically have middle-to-higher wealth indices. This population predominantly lives on the Coast and in Metropolitan Lima. Age distribution is more even across categories than in Cluster 1, although a larger proportion belongs to the 25–29 years group. Higher education levels are common, and Spanish is the dominant language. Ethnically, there is a mix of native and Afro-Peruvian individuals. Testing rates for HIV/AIDS are slightly higher than those in Cluster 1, and media access is nearly universal. Most households are male-headed and most members have health insurance. However, care engagement remains limited.

Cluster 3 is characterized by a high level of HIV/AIDS knowledge, featuring predominantly women living in urban and rural areas. This group tends to have lower wealth indices and is primarily located in the jungle region. Age is evenly distributed across the 15–29 age range, with high school education being the most common form of education. Spanish is widely spoken, with a small portion speaking native languages, and most identifying as having a mixed ethnicity. HIV/AIDS testing rates are low, although access to the media is high. Households are mostly male-headed, and most members have health insurance, although engagement in care is very low.

Cluster 4 demonstrates a medium level of HIV/AIDS knowledge compared to the other groups, with most women and a significant urban presence. Individuals in this cluster often belong to the middle or upper wealth indices and are primarily found on the Coast and in Metropolitan Lima. The age range is relatively balanced, although a larger share is aged between 25 and 29 years. Education levels are generally higher, with many attaining higher education levels. Spanish is the dominant language, and the group largely comprises mixed ethnicities. Testing rates for HIV/AIDS are high, and media access is widespread. Most households are male-headed, and most members have health insurance, although engagement in care remains modest.

The identified clusters show considerable distinctions in terms of HIV/AIDS knowledge, demographic characteristics, and geographic distribution. However, some commonalities can be observed. Health insurance coverage is widespread across all clusters, indicating a relatively broad access to healthcare resources. Despite this, engagement in care remains minimal or limited, suggesting that while coverage exists, active utilization of health services may not be sufficiently promoted or encouraged, potentially affecting access to testing HIV/AIDS-related care. Additionally, access to media is high in each cluster, which implies an ongoing potential to disseminate accurate health information. These factors hint at gaps, not in access, but in proactive engagement with healthcare systems and the media as tools for fostering the dissemination of comprehensive HIV/AIDS knowledge. This alignment between low engagement in care and similar patterns in insurance status across clusters suggests that structural or informational barriers, rather than mere access issues, may play a role in shaping the population’s understanding of and responsiveness to HIV/AIDS-related health practices.

**Figure 9.**
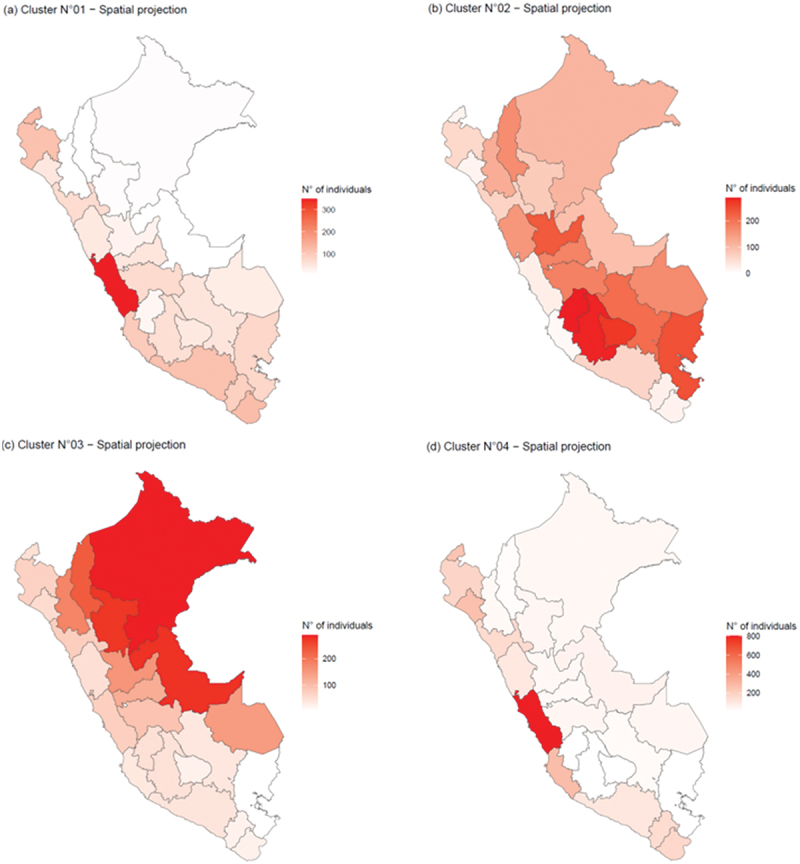
(a) Spatial projection for cluster 1, (b) spatial projection for cluster 2, (c) spatial projection for cluster 3, and (d) spatial projection for cluster 4.

Finally, Figure 9 shows the distribution of individuals across the national plane and highlights the regions of Peru with the highest proportion of respondents per cluster.

The results reveal significant geographical disparities in respondent distribution among the identified clusters. Metropolitan Lima stands out with the highest concentration of individuals in Cluster 1, totaling 309, while other regions, such as Tacna, Arequipa, and Callao, show moderate engagement. In Cluster 2, Ayacucho in Sierra leads with 308 respondents, closely followed by Huancavelica and Apurímac, indicating remarkable awareness in these areas. Conversely, Cluster 3 is marked by a notable number of participants in Lima and Loreto. Cluster 4 demonstrates a strong presence in coastal regions, particularly Lima and Ica, whereas other regions reveal moderate participant levels, primarily from Sierra. These patterns highlight the uneven distribution of knowledge and socioeconomic and health characteristics across the diverse regions of Peru.

## Discussion

We used the SOM algorithm to explore social determinants and HIV/AIDS knowledge among Peruvian youths. By analyzing structural determinants using Kohonen’s neural networks, we identified social clusters linked to the level of HIV/AIDS knowledge, along with sociodemographic, economic, and health characteristics. Our findings have several important implications.

### HIV/AIDS research in Peru

The findings of this study, in which only 33.80% of the respondents demonstrated adequate knowledge of HIV/AIDS, align with those of previous research in Peru. For instance, a study involving women aged 15 years and older found that only 47.8% had a good understanding of HIV, and only 38.9% answered all HIV-related questions correctly. Misconceptions were also prevalent, with 35.2% of women holding incorrect beliefs about HIV transmission [39]. Similarly, another study revealed that although 54.8% of school students in Peru had adequate knowledge of HIV/AIDS, risky sexual behaviors persisted, exhibiting an inverse relationship between knowledge and unsafe sexual attitudes [40].

The results also demonstrate the importance of structural factors in shaping HIV knowledge, which is consistent with previous studies. Our findings indicate that these structural factors, rather than behavioral factors, are relevant to understanding the disparities in HIV knowledge across Peru. Similar trends and findings were observed by Aybar et al. [41], who noted that knowledge of HIV/AIDS was positively associated with certain social determinants of health in the youth population in Peru. Additionally, interventions targeting university students, such as educational programs, have proven effective in significantly increasing knowledge, with students moving from low to moderate awareness levels following participation [42].

Despite progress in some groups, significant knowledge gaps remain, particularly among rural populations and younger individuals, as demonstrated in this study. For instance, research in Tacna, a southern region of Peru, noted that early sexual initiation and low condom use persist, especially in rural areas, despite general awareness efforts [43]. This emphasizes the need for interventions tailored to the specific challenges faced by these vulnerable populations.

### HIV/AIDS research and social determinants of health

Moreover, the SOM analysis differentiated social clusters based on a combination of sociodemographic, economic, cultural, and health-related factors, with a particular focus on HIV/AIDS knowledge. These clusters revealed distinct profiles, allowing for a more detailed understanding of how various groups relate to HIV awareness and risk. This method moves beyond identifying individual factors by recognizing interactions within groups of factors, such as age, sex, income, and education. Numerous studies have confirmed the patterns or outcomes observed in our clusters.

Men were consistently less knowledgeable about HIV/AIDS, with analyses spanning 45 countries indicating that knowledge improved more markedly among young women than among their male counterparts [44]. This aligns with our findings in Clusters 1 and 3, which had a predominantly female composition and showed relatively higher levels of HIV knowledge than the more sex-balanced Clusters 2 and 4. This trend has also been identified in Brazil, where young men aged 18–29 years exhibit riskier sexual behavioral patterns due to lower health knowledge [45]. This age group overlapped with a substantial portion of individuals in Clusters 1 and 2, where knowledge gaps were more prevalent in the persistence of inadequate knowledge within these segments, suggesting the need for targeted interventions to address young men’s awareness of HIV/AIDS risks and prevention. Beyond sex disparities, economic factors were influential, as wealthier individuals in Nigeria demonstrated significantly higher awareness of HIV/AIDS than those with lower wealth levels [46]. This pattern can be compared to our findings, in which Clusters 2 and 4, consisting mainly of urban residents with higher wealth indices, exhibited relatively better awareness, supporting the observation that wealth level influences access to health education and resources.

Geographical disparities were also evident in our study. For instance, individuals from the Sierra region of Peru exhibited lower knowledge levels, reflecting results from Pakistan, where urban residents displayed higher awareness than those in rural provinces [47]. Cluster 1, which largely comprised of individuals from the Sierra region, consistently reflected lower levels of HIV knowledge, reinforcing the geographical disparity highlighted in previous research. The urban-rural divide in access to information and health services remains an important factor affecting awareness levels. Conversely, the connection between education and HIV knowledge is well documented, with studies in Pakistan and Afghanistan highlighting that higher education levels correlate with an increased understanding of HIV transmission and prevention [48]. In our analysis, Cluster 4 stood out for its high educational attainment and correspondingly higher knowledge levels, illustrating how education continues to be a fundamental driver of HIV awareness and aligns with the aforementioned findings.

Similarly, HIV testing was associated with improved knowledge, as observed in Iran, where low testing rates reflected low awareness [49], consistent with our findings. For instance, in Clusters 1 and 3, testing rates were notably low, along with low levels of knowledge, mirroring the challenges identified in the Iranian study and suggesting that barriers to accessing care must be addressed to translate knowledge into effective health actions. Similar associations between prior exposure to HIV/AIDS information and awareness were found in South Sudan, where prior exposure to HIV information was positively correlated with knowledge levels [50]. Clusters 2 and 4 exhibited higher exposure to media and health information sources, which likely contributed to their relatively higher awareness levels. However, engagement in care remained limited, even in these clusters, indicating that exposure to information must be complemented by accessible services to foster consistent healthcare utilization. Moreover, language barriers influence HIV knowledge, with lower awareness among native-language speakers in Peru, a trend also observed in India among foreign-language speakers [51]. In our study, Clusters 1 and 3 had significant portions of individuals who spoke native languages alongside Spanish. Their comparatively lower levels of awareness resonate with the findings from Peru and India, highlighting the ongoing need for language-sensitive health interventions in diverse communities.

Race- and ethnicity-based disparities were also reflected in our data. Afro-Peruvians exhibited knowledge gaps similar to those observed among black and Asian youths in the UK [52]. Clusters 2 and 4, which included individuals of mixed and Afro-Peruvian ethnicities, showed varying levels of awareness, suggesting that ethnic and racial backgrounds continue to influence knowledge disparities. Further, older youths, especially those aged 25–29 years, tended to have better perceptions of HIV, as found in Iran [49]. In Clusters 2 and 4, in which there was a relatively balanced age distribution, individuals aged 25–29 years showed better awareness levels than younger individuals, suggesting that public health efforts could benefit from targeted strategies addressing the younger segments within these clusters to raise their awareness and encourage preventive behaviors.

Considering that health insurance coverage is widespread across all identified clusters, this factor may significantly influence HIV knowledge among the youth. Insured youths access enhanced health services, facilitating education on HIV testing and prevention [53]. Additionally, social and financial support from insurance companies may reduce barriers to healthcare engagement and promote informed health management among youths [54]. Thus, health insurance seems crucial for improving HIV knowledge and health outcomes in this demographic group.

Correspondingly, engagement in care remains minimal in all clusters, reflecting the connection between HIV knowledge and health service utilization. Youths face challenges in navigating healthcare systems, particularly during the transition from pediatric to adult care [53]. Despite reasonable knowledge levels regarding HIV transmission, low testing rates suggest that this does not translate into service engagement [55]. Increased health service use correlates with higher HIV knowledge, as those who are more engaged with healthcare are generally better informed [56]. Clusters 2 and 4, which showed higher knowledge and testing rates, reinforced the importance of promoting healthcare engagement to improve awareness and long-term health outcomes. Thus, targeted interventions focused on accessible and youth-friendly services could bridge the knowledge-behavior gap and enhance engagement in all clusters.

### Traditional approaches and SOM in HIV/AIDS research

The application of SOM in our study provided a useful methodology for examining how social determinants influence HIV/AIDS knowledge among youths, offering distinct advantages over traditional statistical methods. One such benefit is the capacity of SOM to process high-dimensional datasets while preserving the topological relationships among the input variables [17,18]. This is a significant advantage of SOM over traditional statistical methods, which often struggle with high dimensionality, leading to oversimplified models that may overlook complex interactions within the data [12]. This is particularly useful when working with multifaceted data, such as those in public health research, where social, economic, and health-related factors intersect. SOM’s high-dimensional data processing enables researchers to identify patterns and relationships that might provide a comprehensive view of the data [19].

Conversely, traditional epidemiological methods frequently rely on prior assumptions or simplified regression-based approaches to explore associations [12]. Although these models are effective for hypothesis testing, their interpretability may be restricted by linearity constraints and assumptions of variable independence. In contrast, SOM operates unsupervisably, allowing it to reveal the inherently complex, non-linear relationships in multilayered public health data [20]. This attribute is relevant in HIV/AIDS research, where methodologies similar to SOM, such as spatial distribution analysis or dimensionality reduction techniques on social and spatial clusters, can capture complex interactions between factors such as stigma, access to health services, cultural beliefs, and socioeconomic disparities [57]. Approaches that are useful for social and spatial behaviors have proven useful for reducing vulnerability to HIV, highlighting the need to monitor high-case areas and populations to implement effective prevention strategies [58].

Moreover, the SOM approach offers an opportunity to segment populations into clusters that share similar health-related profiles based on a combination of sociodemographic and behavioral characteristics. Unlike traditional methods, which frequently depend on established criteria regarding data distribution or the number of clusters, SOM adapts dynamically to the underlying patterns in a dataset, offering a more flexible means of exploring diverse population structures without rigid constraints [22]. These segments can be further analyzed to tailor public health interventions to the needs of specific groups. In the context of HIV/AIDS research, SOM enables a more nuanced understanding of risk groups, facilitating targeted interventions that can bridge gaps in knowledge or resource access [21]. Previous research has shown that interventions focused on broad demographic groups may miss critical subpopulations that traditional techniques cannot distinguish [22].

Additionally, the flexibility of SOM extends to its adaptability in integrating diverse data types, including spatial, behavioral, and health-related information [59]. This adaptability is especially relevant for HIV/AIDS studies, with similar methodologies as SOM involved in dimension reduction and clustering, where integrating data on testing rates, knowledge, and healthcare access is essential for effective policymaking [60]. These methodologies potentially enable the identification of groups with similar socio-behavioral traits to guide targeted policies, improve resource allocation, and address regional disparities in HIV testing and treatment [61]. SOM’s capacity to analyze these multidimensional relationships simultaneously assists the identification of underexplored dynamics between healthcare accessibility, social support systems, and HIV knowledge or engagement in care [59].

The ability of SOM to visualize and structure high-dimensional relationships without compromising the complexity of the data [62] also enables the design and development of a more sophisticated approach for understanding public health concerns, such as HIV/AIDS. Its unsupervised learning capacity does not merely complement traditional epidemiological methods; it offers a fundamentally different perspective, providing a means to identify emergent properties and hidden clusters that warrant focused public health interventions [62]. This contribution is particularly important for developing targeted and context-specific strategies to address the ongoing challenges in HIV/AIDS prevention and care. SOM’s emphasis on multidimensional clustering and visual representation of clusters offers a unique advantage in visualizing how social determinants interact within population segments [22]. By mapping clusters, SOM can provide actionable insights that inform more focused public health strategies, particularly in identifying hidden HIV clusters and addressing vulnerable youth populations, allowing program planners to prioritize educational resources, support geographically oriented interventions, and improve resource allocation in the complex settings characteristic of low- and middle-income countries [63,64].

### Social clusters for HIV/AIDS interventions in Peru

These findings have important implications for improving HIV/AIDS interventions in Peru. Although core prevention and control measures are being implemented, their impact on reducing new infections remains limited. Key challenges include low political prioritization of prevention, lack of a person-centered approach to health services, barriers to healthcare access, persistent stigma, and inadequate management of information systems. Expanding intervention coverage and enhancing information management are essential for more effective decision-making.

The SOM analysis offers practical insights for refining HIV/AIDS programs. The identified clusters highlight distinct patterns that can guide more targeted interventions. Clusters 2 and 4 share characteristics such as urban residence, middle-to high-wealth levels, and education ranging from high school to higher education. However, Cluster 2 includes individuals with limited HIV/AIDS awareness, balanced sex distribution, and native or Afro-Peruvian ethnicity, whereas Cluster 4 consists of individuals with higher awareness, more women, and mixed ethnic backgrounds. Culturally sensitive programs are necessary to address the unique needs of these populations and ensure inclusivity and equitable access to prevention strategies [65].

For Cluster 4, strategies should be specific but adaptable, considering diverse factors such as age, sex, and wealth group/index. Youths should be actively involved in promoting friendly services and HIV/AIDS education [65]. In Cluster 2, integrating cultural, community, and language elements into prevention strategies is vital, emphasizing ethnic strengths and positive sexual health [66].

Clusters 1 and 3 share attributes, such as a higher proportion of women and very poor individuals. Cluster 1is predominantly composed of households in Sierra, largely of native ethnicity, requiring culturally tailored approaches for indigenous youths [67]. Cluster 3, consisting of households in jungle areas, underscores the need for affordable screening and culturally relevant education on HIV/AIDS and STIs, with a focus on collaborating with community leaders for successful implementation [68].

Identifying these clusters refines resource allocation and improves outreach efforts. By addressing the specific challenges faced by each cluster, policymakers can create targeted strategies to enhance the effectiveness of HIV/AIDS programs, ultimately contributing to a reduction in new infections in Peru.

### Spatial projection of identified social clusters

This study revealed significant variations in the spatial distribution of social clusters among youths in Peru, particularly based on the region of residence. This variability is critical for planning public health interventions aimed at improving HIV/AIDS knowledge while accounting for social and structural factors [69]. The results show a higher concentration of informed individuals in urban coastal areas, which correlates with higher human development and income, compared to Sierra and the jungle regions, where poverty is more prevalent. Disparities in healthcare access, shaped by racial and ethnic factors, are more pronounced in rural areas, where ethnic minorities face greater barriers to services and often lack health insurance, owing to limited resources and geographic isolation [70].

Education is another key factor, particularly for youths aged 15–19 years, who face crucial decisions about their future. Economic disadvantages often lead to early school dropout to work, thus reducing human capital. In contrast, those aged 20–29 years tended to have higher educational attainment and faster learning trajectories [71]. Previous research highlights a significant gap in sexual health awareness in Sierra and the jungle regions, where misconceptions about HIV/AIDS are widespread [72,73]. Similar trends have been observed in Pakistan, where HIV/AIDS knowledge among youths varies significantly by region, with higher awareness in urban and capital areas than in rural provinces [74]. Migration patterns also contribute to social and economic decline in rural Peru, with young people comprising most migratory flows, affecting community renewal, and contributing to urban displacement [75].

Analyzing the spatial distribution of HIV/AIDS knowledge and social determinants can identify priority areas for health interventions in resource-limited settings such as Peru. This can guide resource allocation and policy development to address the burden of HIV and AIDS in specific areas [76]. The study’s reliance on national surveys enhances its representation of the general population, adding to its strength.

### Study limitations

First, the analyzed sample was limited to individuals with HIV/AIDS knowledge, which does not represent the entire population, making a larger sample difficult to achieve nationally. Second, the assessment of knowledge relied on self-perception and specific questions, restricting the depth of analysis, while sophisticated biomarkers are impractical for large samples. Additional limitations include the lack of data on employment and serological status, along with differences in age structure that affect comparability. Moreover, the study’s insufficient sex stratification and cross-sectional design hindered long-term evaluations and causality assessments of the identified factors. Additionally, the inability to incorporate broader structural- or community-level factors, such as cultural beliefs, healthcare engagement, and peer influence, owing to their absence from the survey data, limits the analysis. This restriction hinders a comprehensive understanding of the social dynamics surrounding HIV/AIDS knowledge among youths in Peru. There is a need for future studies to address these gaps and for surveys to include relevant variables that capture these dimensions more effectively.

Although using Kohonen’s network was beneficial, there is room for improvement in information visualization, dimensionality reduction, and integration with other data mining methods. Experiments with different vectorizers and distance metrics can provide deeper insights. Finally, expanding the scope of the study to include other data sources for mapping key populations can enhance the identification of HIV/AIDS and deepen our understanding of the epidemic in Peru. Implementing alternative sampling techniques to oversample low-density areas is crucial to increase the reliability of future survey results.

## Conclusions

To conduct an exploratory assessment of our primary data to identify clusters of knowledge about HIV/AIDS and its social determinants at the national level, we designed an unsupervised learning method based on SOMs. This enabled the identification of the patterns and behaviors of the youth population in Peru, quantifying and characterizing social clusters in the country.

The proposed methodology identified groups of youths with shared characteristics. This makes it possible to discern the degree of concordance among inhabitants regarding specific profiles and behaviors based on cultural, economic, social, and health factors. The implementation of an SOM network may have proven effective in detecting the fragmentation of behaviors and profiles among Peruvian youth, considering the heterogeneity of the identified clusters. Hence, the application of SOM in public health can effectively support current measures.

From a technical perspective, SOMs have demonstrated their efficacy as valuable tools for analyzing social and demographic databases. However, any revealed association requires thorough scrutiny using conventional statistical methods. Nevertheless, a methodology capable of offering preliminary insights into the current body of literature on HIV/AIDS in Peru is valuable in this field of study.

Furthermore, Kohonen’s map emerges as an effective platform for visualizing high-dimensional data, allowing for an optimal division of prototypes generated from observations into discernible groups. Through an approximate visual presentation of clusters and low computational cost, this method facilitates the understanding of the relationship between independent variables and the outcomes of cluster configuration, enabling the determination of correlation levels among factors and the shape of the map, and offering a clear overall description of local relationships within the data.

Our findings would enable the development of more targeted interventions and stronger policies based on identified profiles, with a focus on vulnerable groups, such as the Peruvian youth, bringing these focal points to national attention. Although the 2023 implementation of the Technical Health Standard for Combined HIV Prevention marks a significant step in HIV/AIDS prevention and control, its current reach has not significantly reduced the number of new infections. Efforts must be expanded and improved to achieve a greater decline and a sustained downward trend [9]. Building on this framework with clustering results, it is essential to broaden the scope of key interventions and improve information management to guide decision-making, particularly in addressing the needs of younger populations that have been insufficiently considered in existing strategies. The cluster analysis is also beneficial for identifying which segments of the youth population require specific attention in terms of primary and secondary prevention activities, reducing the stigma and social barriers that hinder access to health services. This supports the need for more precise prevention strategies within the 2023 program, including improved communication, multisectoral coordination, a formal combined prevention model, and the integration of fixed, mobile, and community-based services. These measures can be adapted based on the insights from the SOM analysis, which identified specific demographic profiles and areas for focused intervention. SOM network analysis can be a valuable tool for researchers and policymakers to assess equity issues and optimize resource allocation for social and health services, increasing the potential for effective implementation and engagement with current programs.

Finally, further studies based on artificial intelligence techniques are recommended, given their potential benefits in public health and policies [77].

## Data Availability

The data presented in this study are available upon reasonable request from the corresponding author.
